# Effects of Chemical and Radiation Sterilisation on the Biological and Biomechanical Properties of Decellularised Porcine Peripheral Nerves

**DOI:** 10.3389/fbioe.2021.660453

**Published:** 2021-06-03

**Authors:** James D. R. Holland, Georgina Webster, Paul Rooney, Stacy-Paul Wilshaw, Louise M. Jennings, Helen E. Berry

**Affiliations:** ^1^School of Biomedical Sciences, Faculty of Biological Sciences, University of Leeds, Leeds, United Kingdom; ^2^School of Mechanical Engineering, Faculty of Engineering, University of Leeds, Leeds, United Kingdom; ^3^National Health Service Blood and Transplant (NHSBT) Tissue and Eye Services, Liverpool, United Kingdom; ^4^School of Pharmacy and Medical Sciences, Faculty of Life Sciences, University of Bradford, Bradford, United Kingdom

**Keywords:** peripheral nerves, decellularisation, sterilisation, collagen type IV, biomechanical properties

## Abstract

There is a clinical need for novel graft materials for the repair of peripheral nerve defects. A decellularisation process has been developed for porcine peripheral nerves, yielding a material with potentially significant advantages over other devices currently being used clinically (such as autografts and nerve guidance conduits). Grafts derived from xenogeneic tissues should undergo sterilisation prior to clinical use. It has been reported that sterilisation methods may adversely affect the properties of decellularised tissues, and therefore potentially negatively impact on the ability to promote tissue regeneration. In this study, decellularised nerves were produced and sterilised by treatment with 0.1% (v/v) PAA, gamma radiation (25–28 kGy) or E Beam (33–37 kGy). The effect of sterilisation on the decellularised nerves was determined by cytotoxicity testing, histological staining, hydroxyproline assays, uniaxial tensile testing, antibody labelling for collagen type IV, laminin and fibronectin in the basal lamina, and differential scanning calorimetry. This study concluded that decellularised nerves retained biocompatibility following sterilisation. However, sterilisation affected the mechanical properties (PAA, gamma radiation), endoneurial structure and basement membrane composition (PAA) of decellularised nerves. No such alterations were observed following E Beam treatment, suggesting that this method may be preferable for the sterilisation of decellularised porcine peripheral nerves.

## Introduction

Peripheral nerve injuries are frequent in the population; a 2014 study by Castillo-Galvan et al. found an incidence rate of 1.12% (Castillo-Galván et al., 2014). Due to the relatively young patient demographic (mean age of incidence 27 years), peripheral nerve injuries are associated with a high economic burden from associated treatment costs and loss of productivity. Peripheral nerve injuries are typically the result of traumatic injury (e.g., excessive tension, crushing or partial/complete transection); such injuries have poor levels of functional recovery without therapeutic intervention (Sullivan et al., 2016). Current therapies for the repair of peripheral nerve injuries are not ideal and have several disadvantages. Harvesting of autografts, the current gold standard therapy, causes donor-site morbidity, loss of a sensory nerve and may not be suitable for the repair of larger defects due to the quantity of tissue required (Wolford and Stevao, 2003). The efficacy of synthetic nerve guidance conduits is limited to defects which are shorter than a critical length (approximately 3 cm); this is believed to be due to a lack of microscale directional guidance and/or chemotrophic cues which aid cellular regeneration (Isaacs and Browne, 2014).

Previous research in the United States resulted in the development of a decellularised human peripheral nerve, the Avance^®^ nerve graft, which is currently in clinical use (Moore et al., 2011). Decellularised porcine peripheral nerves represent an alternative graft material due to structural similarities to human nerves (Zilic et al., 2015). The use of porcine tissue as a source material has significant advantages in terms of availability and consistency of tissue quality. A decellularisation process for porcine peripheral nerves using low concentration sodium dodecyl sulphate (SDS) and hypotonic buffers was previously developed (Zilic et al., 2016). Porcine decellularised nerves were found to be biocompatible, retain an extracellular matrix (ECM) structure similar to that of human peripheral nerves and support the growth of Schwann cells in both *in vitro* and *in vivo* environments (Unpublished data and Zilic et al., 2016).

In order to be used clinically, medical devices derived from xenogeneic sources should be sterilised to meet the level stipulated in standard BS EN 556-1 (BSI, 2001). BS EN 556-1 states that a sterilisation method should be able to ensure that less than 1 in 1,000,000 devices sterilised by a defined process may contain a viable pathogen. Any sterilisation method applied should not adversely affect the functional properties of the tissue scaffold, as any such changes may compromise tissue regeneration.

Sterilisation of biological materials (including tissue scaffolds) can be carried out using methods such as ethylene oxide gas and ionising radiations including gamma radiation and electron beam (E Beam; Bernhardt et al., 2015). Accepted parameters for the effective use of these methods are detailed in ISO standards, such as the BS EN ISO 11137 standard for sterilisation with ionising radiation and BS EN ISO 11135 for sterilisation with ethylene oxide (ISO, 2014, 2015a,b). Other methods have also been studied and are regarded as effective sterilants: one such method is the use of peracetic acid (PAA) solution, an oxidising agent which may be used with medical devices derived from xenogeneic source materials according to BS EN ISO 14160 (ISO, 2011). The efficacy of PAA with regard to tissue scaffold sterilisation has been studied in combination with a range of solvents; however, PAA has not been as widely characterised as more established sterilisation methods have been (Hodde and Hiles, 2002). Published studies have demonstrated that sterilisation of tissue scaffolds may alter the mechanical properties, ECM structure, biocompatibility, and surface chemistry of biological materials, through a variety of mechanisms, although some studies are contradictory (Table 1). The penetrative ability of ionising radiation makes it a good candidate for the terminal sterilisation of tissue scaffolds; this is sterilisation of a graft or device within the final packaging, with no further handling or transfer necessary prior to clinical use (Lambert et al., 2011). Following treatment in PAA solution (or any liquid sterilant) a tissue scaffold must be aseptically transferred to final packaging according to parameters detailed in BS EN ISO 13408, usually in a high-grade clean room (ISO, 2015a,b). This provides an opportunity for contamination of the scaffold to occur post-sterilisation (Bernhardt et al., 2015). The use of methods enabling terminal sterilisation are therefore preferable.

**TABLE 1:** Summary of sterilisation methods and known adverse effects when used with biological materials.

Sterilisation method	Possible adverse effects
Heat (e.g., steam, dry heat)	Generally considered unsuitable for ECM-based scaffolds, due to exceeding the denaturation temperature of collagen (approximately 65°C) (Bozec and Odlyha, 2011)
Oxidising agents (e.g., PAA, hydrogen peroxide)	Denaturation/degradation of molecular ECM components, probably through oxidative reactions, with associated disruption to structural properties and capacity to support remodelling (Scheffler et al., 2008)
Alkylating agents (e.g., ethylene oxide)	Incomplete aeration can result in the deposition of residual ethylene oxide and by- products (e.g., ethylene chlorohydrin, ethylene glycol), linked to acute and chronic toxicities and the induction of an inflammatory response. Also linked to mechanical alterations (Lomas et al., 2001; Bernhardt et al., 2015)
Ionising radiation (e.g., gamma radiation, E Beam)	Dependent on conditions. Induces formation of cross-links if tissue is in a wet state, leading to increases in mechanical parameters such as stiffness or mechanical strength and preserves ECM architecture. If tissue is in a dry state, scission of the collagen chains predominates and causes degradation of the ECM and negatively affects mechanical properties (Cao et al., 2013)

The identification of a sterilisation method that results in sufficient bioburden reduction whilst minimising damage to the scaffold is key to facilitating clinical translation of decellularised porcine peripheral nerves. Therefore, this study aimed to determine the effects of three sterilisation methods on the structure, biocompatibility, biomechanical properties and biochemical composition of decellularised porcine peripheral nerves. The selected sterilisation methods were gamma irradiation, electron beam irradiation, and peracetic acid. Although ethylene oxide is a well recognised terminal sterilisation method for healthcare products, it was excluded from this study due to the following reasons; potential toxicity of chemical by-products, necessity of presenting the products in a dry state, to facilitate efficient gas penetration and then aeration/desorption post-ethylene oxide exposure, and preliminary investigations (unpublished) revealing impairment of scaffold rehydration potential and loss of ECM structural integrity following lyophilisation.

## Materials and Methods

Unless otherwise stated, all reagents used during the study were obtained from Sigma-Aldrich (Dorset, United Kingdom), VWR International (Lutterworth, United Kingdom) or Fisher Scientific (Leicestershire, United Kingdom).

### Dissection of Porcine Peripheral Nerves

The sciatic nerve plexus was dissected from the hind legs of Large White pigs, aged approximately 6 months, obtained within 24 h of slaughter from a local abattoir (Leeds, United Kingdom), as previously described (Zilic et al., 2016). The tibial and peroneal nerves were dissected and cleaned of excess connective tissue. Each nerve was washed in PBS (Oxoid; pH 7.4) with gentle agitation three times, before being stored at –80°C until needed.

### Decellularisation

Segments of thawed nerve were decellularised according to a method adapted from Zilic et al. (2016). All washes were conducted with agitation at a speed of 110 rpm (PSU-10i Orbital shaking platform, Grant Instruments, United Kingdom), unless otherwise stated. Briefly, nerve segments (30–40 mm in length) were washed in antibiotic solution (0.05 mg/mL vancomycin hydrochloride, 0.5 mg/mL gentamycin sulphate, 0.2 mg/mL polymyxin B) for 30 min at 37°C, then 200 mM EDTA (pH 7.2–7.4) for 24 h at 4°C. Each segment was then washed in hypotonic buffer (10 mM Tris-HCL, 2.7 mM EDTA, 10 KIU/mL aprotinin; pH 8.0–8.2) for 24 h at 42°C, followed by hypotonic buffer containing 0.1% (w/v) SDS for 24 h at 42°C. Four washes in PBS (3 × 30 min, 1 × 16 h) at 42°C and one 72 h wash in PBS-EDTA solution (2.7 mM EDTA, 10 KIU/mL aprotinin; pH 7.2–7.5) at 4°C. The segments were then incubated twice in nuclease solution (50 mM Tris, 1 mM MgCl_2_.6H_2_O, 1 U/mL Benzonase [Merck KGaA, Germany]; pH 7.5–7.7) for 3 h at 37°C with agitation (50 rpm). Finally, the nerve segments were sequentially washed in PBS (16 h), hypertonic buffer (50 mM Tris, 1.5 M NaCl; pH 7.5–7.7; 24 h) and PBS (3 × 30 min) at 42°C.

### Sterilisation

Peripheral nerve segments were sterilised using one of three methods; peracetic acid (PAA) solution, gamma radiation or electron beam.

#### Peracetic Acid

A group of decellularised nerve segments (*n* = 30) were treated with peracetic acid in PBS, following the method developed by Lomas et al. (2003). Briefly, the nerve segments were incubated in 0.1% (v/v) PAA in PBS solution (pH 7.2, 30 mL/segment) at room temperature for 3 h with gentle agitation, then washed three times in PBS (pH 7.4) for 30 min each.

#### Gamma Radiation

A group of decellularised nerve segments (*n* = 30) were suspended individually in 3 mL PBS (within polystyrene tubes). The nerves were subjected to gamma irradiation at a dose of 25–28 kGy (Foss Therapy Model 812 cobalt-60 self contained high dose rate gamma irradiator; Dalton Cumbrian Facility, University of Manchester). The temperature within the irradiation chamber did not exceed 35°C.

#### Electron Beam Irradiation

A further group of decellularised nerve segments (*n* = 30) were suspended individually in 3 mL PBS (within polystyrene tubes), and treated with an electron beam, receiving a dose of approximately 33–37 kGy (10 MeV, 15 kW, Horizontal Beam; STERIS, Daventry, United Kingdom).

### *In vitro* Evaluation of Scaffold Biocompatibility

The biocompatibility of the sterilised decellularised nerves was determined by contact cytotoxicity experiments using two distinct cell lines.

#### Cell Culture

Cells were sourced from the Health Protection Agency (HPA), formerly European Collection of Authenticated Cell Cultures. L929 murine fibroblasts were cultured in Dulbecco’s modified essential media (DMEM), supplemented with 10% (v/v) foetal bovine serum (FBS), 2 mM L-glutamine, 100 U/mL penicillin and 100 μg/mL streptomycin, at 37°C in 8% (v/v) CO_2_ in air. Baby hamster kidney (BHK-21 STRAIN 31) cells were cultured in Glasgow’s modified essential media (GMEM), supplemented with 5% (v/v) FBS, 10% (w/v) tryptone phosphate broth (TPB), 2 mM L-glutamine, 100 U/mL penicillin and 100 μg/mL streptomycin, at 37°C in 8% (v/v) CO_2_ in air.

#### Contact Cytotoxicity Testing

Segments of decellularised and sterilised decellularised nerve (5 mm in length, *n* = 3 for each group) were placed into individual wells of six well culture plates, and attached to the surface using surgical closure strips. Culture wells containing only cyanoacrylate adhesive or surgical closure strips were included as positive and negative controls for cytotoxicity, respectively. L929 and BHK cells were then seeded at a concentration of 250,000 cells/mL into each of the test and control wells, and incubated (37°C, 8% v/v CO_2_ in air) for 48 h. The media was aspirated from each well, the cell sheets were washed three times using PBS and subsequently fixed using 10% (v/v) neutral buffered formalin (NBF) for 10 min. The cell sheets were stained using Giemsa stain for 5 min, before rinsing with distilled water. The wells were examined using an inverted microscope to determine changes in cell growth and morphology, all images were captured digitally.

### Histological Evaluation of ECM Structure

Segments of decellularised and sterilised decellularised porcine peripheral nerve (*n* = 3 for each group) were fixed using 10% (v/v) NBF and embedded into paraffin wax. Sections of nerve (5 μm) were stained with haematoxylin and eosin (H&E) to evaluate the overall structure of the ECM, and Sirius red with Miller’s to visualise the collagenous (predominantly collagen type I) and elastic components. Sections were viewed under Köhler illumination or circularly polarised light (Sirius red sections only), all images were captured digitally.

### Immunohistochemical Labelling of Key Basement Membrane Components

Antigen retrieval was carried out on paraffin embedded sections by incubation in proteinase K solution (ThermoFisher Scientific) at room temperature for 20 min. Hydrogen peroxide solution (3%; v/v) was used to quench endogenous peroxidase activity. Antibody labelling for specific epitopes using a 3, 3’-diaminobenzidine (DAB) peroxidase-mediated detection system was carried out against laminin (polyclonal, Sigma), and collagen type IV (monoclonal, DAKO). Antibody labelling for fibronectin (polyclonal, DAKO) was detected via a secondary antibody tagged with AlexaFluor-488 (polyclonal, Invitrogen). The slides were viewed using normal Köhler illumination for the DAB detection system or a specific wavelength of 488 nm for fluorescently labelled sections, all images were captured digitally.

### Evaluation of Biomechanical Properties

Native, decellularised (negative control) and sterilised decellularised nerves (*n* = 6 for each group) were subjected to uniaxial tensile testing to failure. A further two positive control groups were added: decellularised nerves incubated in 10% (v/v) NBF were included as a control group representing sample crosslinking, whilst decellularised nerves incubated overnight in 1 M sodium hydroxide (NaOH) were included as a control representing extensive denaturation of proteins within the sample. A gauge length of 10 mm was used; the width and thickness of each sample was determined using calipers and a thickness gauge (Mitutoyo), respectively. Individual samples were loaded without tension into custom made rig, which was then loaded onto an Instron 3,365 mechanical test machine. The samples were subjected to tensile testing to failure with an extension rate of 10 mm⋅min^–1^. Failure was defined as the point at which the maximum load was sustained by the sample. Load extension curves were produced directly from the recorded data, whilst stress-strain curves were produced for each sample by combining the acquired load-extension data with the sample dimensions. The data was plotted as both load-extension and stress-strain curves, from which the values of key mechanical parameters were derived. The value corresponding to the failure point on the load-extension curve was taken as the maximum load at failure. The value corresponding to the failure point on the stress-strain curve was taken as the ultimate tensile strength (UTS), whilst the gradient of the linear region was determined to give the Young’s Modulus.

### Quantification of Collagen and Denatured Collagen Content

Native, decellularised and sterilised decellularised nerves (*n* = 6) were subjected to biochemical assays to quantify collagen and denatured collagen content. To quantify collagen, acid-hydrolysed tissue samples were assayed for hydroxyproline content based on an established method (Edwards and O’Brien, 1980). Denatured or damaged collagen content was assessed using enzymatic digestion of tissues using α-chymotrypsin (Type II, bovine pancreas, Sigma) followed by acid hydrolysis of the supernatant. Hydroxyproline levels were determined using linear regression of a trans-4-hydroxy-L-proline standard curve. Collagen content was estimated using a conversion factor of 7.52 (Cross et al., 1973).

### Differential Scanning Calorimetry

The thermal stability of collagen within native, decellularised and sterilised decellularised nerves (*n* = 6 for each group) was assessed by differential scanning calorimetry (DSC) using a Q2000 differential scanning calorimeter. Samples were placed into hermetically sealed aluminium containers. The temperature was increased at a constant rate of 4°C/min, from 15–130°C. The denaturation transition temperature of each sample, defined as the temperature at which maximum heat flow was observed, was recorded.

### Data Analysis

Data was analysed using the GraphPad Prism software. All data is presented as mean ± 95% confidence intervals. The datasets were analysed using one-way ANOVA, with Tukey’s *post hoc* analysis was used to determine the significance of differences seen between groups. A significant difference was defined as a *p*-value of <0.05.

## Results

### *In vitro* Evaluation of Scaffold Biocompatibility

**FIGURE 1:**
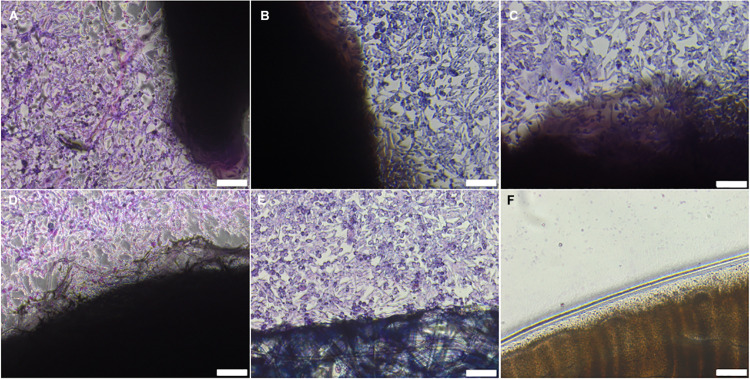
BHK cells cultured with **(A)** non-sterilised, **(B)** PAA-treated, **(C)** gamma irradiated or **(D)** E Beam treated decellularised nerve. **(E)** Surgical closure strip and **(F)** cyanoacrylate adhesive were used as negative and positive controls, respectively. The location of the tissue samples is indicated by the dark shadow regions. Cells were stained using Giemsa’s stain. Images were acquired using a ×10 objective, scale bars = 100 μm.

**FIGURE 2:**
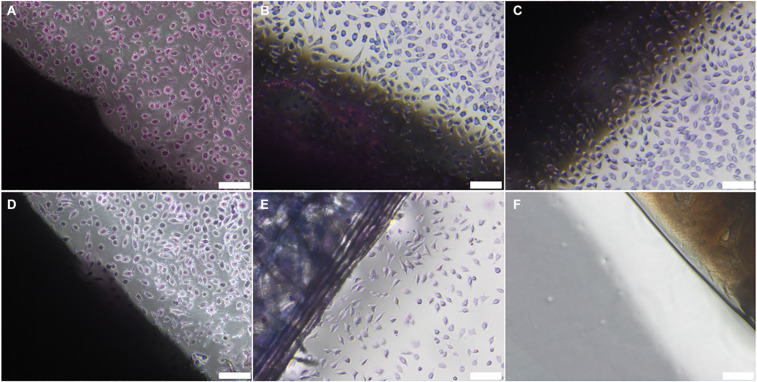
L929 cells cultured with **(A)** non-sterilised, **(B)** PAA-treated, **(C)** gamma irradiated or **(D)** E Beam treated decellularised nerve. **(E)** Surgical closure strip and **(F)** cyanoacrylate adhesive were used as negative and positive controls, respectively. The location of the tissue samples is indicated by the dark shadow regions. Cells were stained using Giemsa’s stain. Images were acquired using a ×10 objective, scale bars = 100 μm.

No evidence of cytotoxicity was observed in BHK or L929 cells cultured with non-sterilised, PAA-treated, gamma irradiated or E beam treated decellularised nerves (Figures 1, 2). The cellular populations were similar to those seen in the negative control wells containing surgical closure strips; widespread death or morphological change was not observed in either BHK or L929 cells. No cells were observed in wells containing the positive control, cyanoacrylate adhesive (Figures 1, 2).

### Histological Evaluation of ECM Structure

**FIGURE 3:**
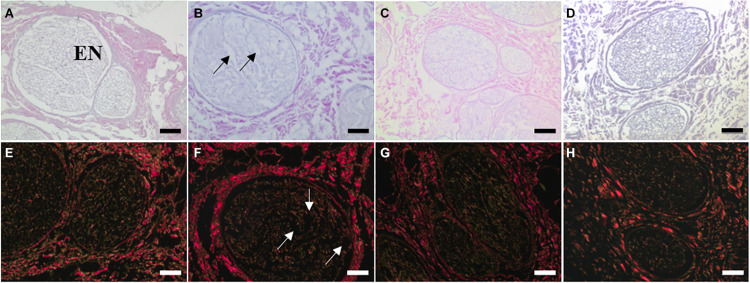
Sections of **(A,E)** non-sterilised, **(B,F)** PAA-treated, **(C,G)** Gamma irradiated, and **(D,H)** E Beam treated decellularised nerve stained with **(A–D)** H&E and **(E–H)** Sirius Red. Key ECM structures (e.g., endoneurial tubules, perineural (PE) lamellar structure) appeared to be well conserved following treatment with ionising radiation, however some structural disruption was evident in PAA-treated sections. This was particularly noticeable when considering the fine structure of the endoneurial tubules (EN). Structural disruption indicated by arrows. Images were acquired using a ×10 objective, scale bars = 100 μm.

The H&E and Sirius Red staining demonstrated that key ECM structures, such as the endoneurial tubes and lamellar layers of the perineurium, were preserved following treatment of decellularised nerves with ionising radiation (Figure 3). In contrast, treatment of decellularised nerves with PAA appeared to cause some structural disruption in each of the recognised ECM regions of the nerve. The region which appeared to be most affected was the endoneurium, where extensive alteration to the structure of the individual tubules was evident.

### Immunohistochemical Labelling of Key Basement Membrane Components

**FIGURE 4:**
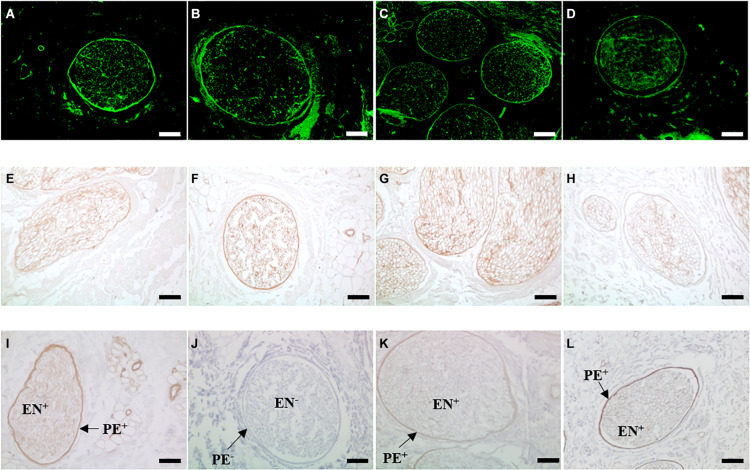
Sections of **(A,E,I)** non-sterilised, **(B,F,J)** PAA-treated, **(C,G,K)** Gamma irradiated and **(D,H,L)** E Beam-treated decellularised nerve immunohistochemically labelled for the presence of the key basement membrane components **(A–D)** fibronectin, **(E–H)** laminin and **(I–L)** collagen type IV. Positive staining for laminin and collagen type IV is indicated by the areas of intense brown colour. Staining for each component is localised to the basement membrane regions of the perineurium and endoneurium. Fibronectin and laminin appeared to be conserved following all sterilisation treatments. Collagen type IV labelling was not present in PAA-treated decellularised nerve (labelled PE- and EN-, but was retained in the perineurium (PE+) and endoneurium (EN+) nerves treated with ionising radiation, at a reduced intensity. Images were acquired using a ×10 objective, scale bars = 100 μm.

Antibody labelling indicated that fibronectin and laminin were retained following treatment with PAA, gamma radiation or E beam (Figure 4). Labelling was highly specific, localised to the basement membrane regions of the perineurium and endoneurium. Labelling was particularly concentrated in the dense lamellar layers of the perineurium. Collagen type IV appeared to be preserved following treatment of decellularised nerves with ionising radiation, although the signal intensity appeared to be slightly reduced in intensity. Decellularised nerves treated with PAA displayed no positive staining for collagen type IV in either the perineurium or endoneurium.

### Evaluation of Biomechanical Properties

**FIGURE 5:**
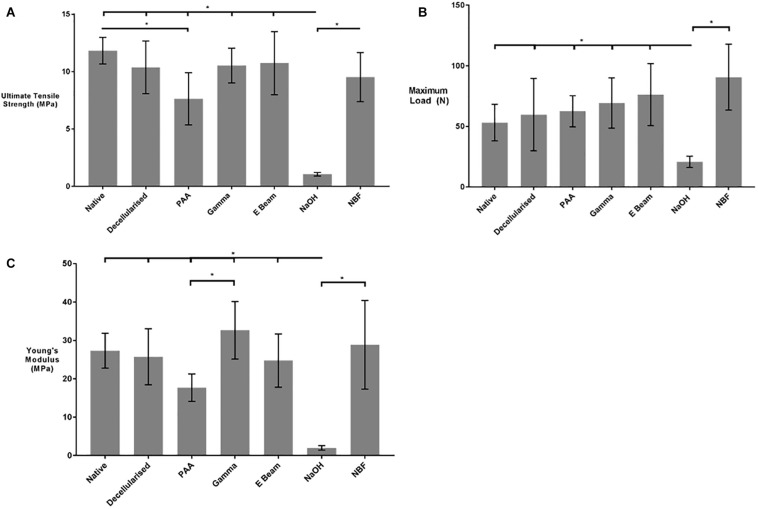
**(A)** Ultimate Tensile Strength (UTS), **(B)** Maximum load at failure and **(C)** Young’s Modulus of nerves in different treatment groups. PAA-treated decellularised nerves were found to have a significantly reduced UTS in comparison to native tissue. No significant differences were found in the maximum load between the sterilisation treatment groups. The Young’s Modulus of gamma irradiated decellularised nerve was significantly greater than that of PAA-treated decellularised nerves. No differences were found the Young’s Modulus of non-sterilised decellularised nerves and any of the sterilisation treatment groups. Decellularised nerves treated with NaOH and NBF were included as controls. Data analysed by one-way ANOVA. Data represents mean ± 95% confidence intervals (*n* = 6). Significant differences are denoted by *(*p* < 0.05).

Uniaxial tensile testing was performed to determine any changes in the biomechanical properties of decellularised nerves following sterilisation. Treatment with PAA resulted in a significant decrease in the ultimate tensile strength of decellularised nerves, when compared to native or non-treated decellularised nerves, whereas no significant difference was seen in those grafts treated with gamma radiation and E Beam (Figure 5A). In contrast, no differences in maximum load at failure were seen following any of the sterilisation treatments (Figure 5B). The Young’s Modulus of decellularised nerves treated with PAA was significantly lower than the Young’s Modulus of decellularised nerves treated with gamma radiation; however, none of the sterilisation treatments caused a reduction in Young’s Modulus when compared to native or untreated decellularised nerves (Figure 5C). Denaturation of ECM proteins by treatment with NaOH (positive control) resulted in a significant reduction in all measured parameters.

### Quantification of Collagen and Denatured Collagen Content

The collagen and denatured collagen content were determined through the use of colourimetric assays. The hydroxyproline content of hydrolysed tissue or α-chymotrypsin digests was determined, and a conversion to a representative collagen content achieved through calculation using a previously determined factor of 7.52 (Cross et al., 1973). No significant difference was found between the collagen content or denatured collagen content of any of the treatment groups (Table 2). Decellularised nerves treated with NaOH (positive control) had an approximately 30-fold higher denatured collagen content than all other treatment groups.

**TABLE 2:** Collagen content of nerves in different treatment groups, calculated by conversion of measured hydroxyproline content using a known factor.

Group	Native	Decellularised	PAA	Gamma	E Beam	NaOH
Collagen content (μg.mg^–1^)	284.8 (±70.7)	334 (±117.0)	353 (±46.6)	343.2 (±141.1)	347.7 (±64.4)	334.6 (±90)
Denatured collagen content (μg.mg^–1^)	5.32 (±2.26)	7.32 (±2.62)	3.65 (±1.37)	7.38 (±3.59)	7.48 (±0.91)	248 (±45.95)

*Data represents mean ± 95% confidence intervals. Data analysed by one-way ANOVA. No significant differences were found between treatment groups (p > 0.05).*

### Differential Scanning Calorimetry

**FIGURE 6:**
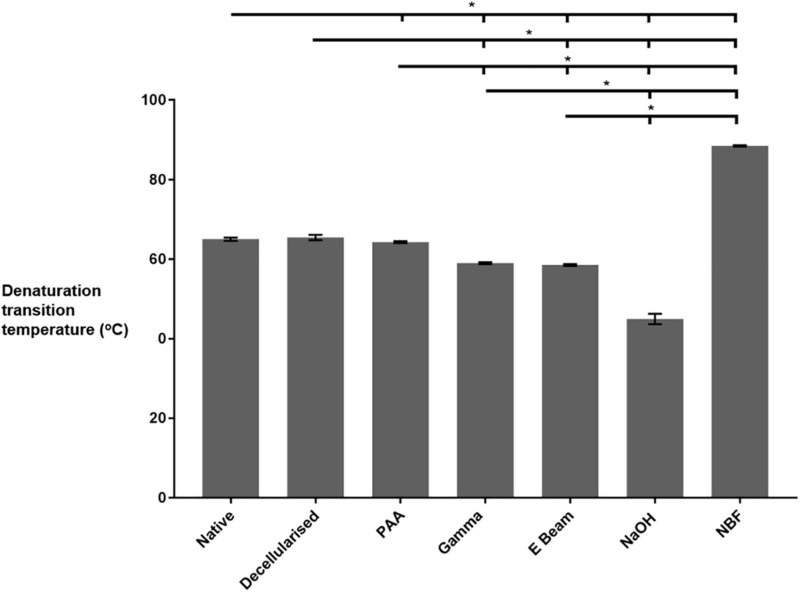
Thermal stability of nerves in different treatment groups, as determined by DSC analysis. Decellularised nerves sterilised by treatment with PAA, gamma radiation or E Beam all had a significantly reduced denaturation transition temperature (defined as peak heat flow) in comparison to non-sterilised decellularised nerves. Data analysed by one-way ANOVA. Data represents mean ± 95% confidence intervals (*n* = 6). Significant differences are denoted by *(*p* < 0.05).

The thermal stability of the collagen within the nerves was analysed using DSC and the collagen denaturation transition temperature was recorded (Figure 6). Sterilisation of decellularised nerves with both gamma radiation and electron beam caused a reduction in denaturation transition temperature, in comparison to native and non-sterilised decellularised nerves. In contrast, the denaturation transition temperature of decellularised nerves treated with PAA was found to be significantly reduced in comparison to non-sterilised decellularised nerves, but not native nerves. Treatment with NaOH (positive control for protein denaturation) resulted in a reduced denaturation transition temperature in comparison to all other groups, and nerves treated with NBF (positive control for peptide cross-linking) were found to have an increased denaturation transition temperature when compared to all other groups.

## Discussion

There is a need for a novel graft material to promote and support functional regeneration across peripheral nerve defects; this is underlined by the inherent limitations of the current clinical options, such as poor efficacy in large defect lengths and donor site morbidity with autograft use (Moore et al., 2009; Gao et al., 2013). Decellularised nerve grafts derived from xenogeneic sources may provide an alternative to autografts for peripheral nerve repair, since xenogeneic bioscaffolds can present many of the advantages of unprocessed autografts, such as a native-like ECM structure, cell attachment motifs and chemotrophic cues, and appropriate biomechanical properties (Crapo et al., 2011; Badylak, 2014; Zilic et al., 2015).

To reduce the risk of a foreign body-related infection following implantation, grafts should be rendered sterile. For decellularised xenografts, considered to be medical devices, EN 556-1 is the European standard specifying requirements for designating a device as terminally sterilised, which should be applied unless the devices are shown to be unable to withstand a terminal sterilisation processes. Sterilisation methods have been known to degrade ECM components and structure through mechanisms such as cross- linking, chain scission, oxidation and heat-mediated damage, some of which are tissue type-dependent (Singh et al., 2016). The choice of a sterilisation method for a graft should therefore be guided by assessment of the specific tissue in question; in particular, evaluation of those biological and/or physical properties considered crucial for desired clinical function of the graft.

Currently, two decellularised human nerve grafts have undergone multicentre clinical evaluations; a non-commercial nerve graft for sensory nerve defects in China (He et al., 2015) and the commercial Avance^®^ nerve graft from Axogen in the United States (Kasper et al., 2020; Safa et al., 2020). Both are sterilised with 25 kGy gamma radiation; however, it is unclear why this method was selected, and limited data are available regarding the effects of the sterilisation process in isolation on the properties of the decellularised nerve tissue. The results of initial trials indicated that both grafts were safe, did not elicit an adverse host immune response, and revealed that (at least partial) functional restoration could be achieved in short to moderate length (0.5–5 cm) defects (He et al., 2015; Kasper et al., 2020; Safa et al., 2020). These initial data suggest that sterilisation with ionising radiation does not impair functional properties of decellularised nerves.

Gamma sterilisation has historically been used for the sterilisation of both unprocessed allografts and decellularised allo/xenografts; examples include decellularised human dermis (Gabriel and Maxwell, 2018), allogeneic bone grafts (Loty et al., 1990; Vargas and Caton, 2009), and allogeneic amniotic membrane (Singh et al., 2004, 2013). However, the potential negative effects of processing with gamma radiation are also well documented; as one example, gamma irradiation (12 kGy) was demonstrated to induce disruption to the fine ECM structure, and cause a reduction in the ultimate tensile strength, of a decellularised human dermal graft (Gouk et al., 2008). The specific conditions used (e.g., dose level, or whether the tissue is dehydrated prior to irradiation) are increasingly recognised as important determinants of the gamma irradiation effects on tissue (Smith and Kearney, 1996; Kajbafzadeh et al., 2013; Singh et al., 2016; Herbert et al., 2017). Screening of a range of sterilisation methods with differing modes of action (and therefore presenting differing advantages and disadvantages) was therefore considered to be the best approach for the identifying a suitable sterilisation method for the decellularised porcine peripheral nerves, intended to be an “off-the-shelf” clinical product that retained key biological and physical tissue properties.

The tissue properties identified as potentially crucial for the optimal function of decellularised nerves *in vivo* included; the ECM structure and basement membrane composition of the endoneurium, which provides chemotrophic and physical guidance to infiltrating Schwann cells and neurons, the structure of the perineurium (an important determinant of peripheral nerve mechanical properties), and the retention of a native-like Young’s Modulus to ensure regenerating neurons residing within the graft are not subject to excessive tensile mechanical forces (Topp and Boyd, 2006, 2012). Validation of the sterilisation efficacy (i.e., bioburden reduction) was beyond the scope of this study.

PAA solution, gamma radiation, E Beam and ethylene oxide were initially selected as candidate sterilisation methods due to their proven efficacy in terms of bioburden reduction, demonstrated either through the existence of a relevant standard (e.g., ISO 11137 for ionising radiations), or via prior data indicating that a sterility assurance level equivalent to that stipulated in BS EN 556-1 can be achieved through the application of a given method (e.g., ISO 14160 and ISO 11135). In addition, there is an established evidence base for the use of PAA (e.g., decellularised human dermis), gamma radiation (e.g., allogeneic amniotic membrane, decellularised human dermis, decellularised porcine superflexor tendon), and E Beam (e.g., decellularised porcine superflexor tendon, human flexor digitorum superficialis tendon) indicating possible compatibility with soft tissue grafts (Singh et al., 2004, 2016; Wei et al., 2013; Marsit et al., 2014; Hogg et al., 2015; Edwards et al., 2017; Gabriel and Maxwell, 2018). Ethylene oxide was considered for inclusion as a candidate sterilisation method due to its efficacy (in terms of bioburden reduction) and previous history of use in tissue banking, particularly with bone allografts (Prolo et al., 2013). However, the use of ethylene oxide has been associated with the induction of a range of detrimental effects to scaffolds, including compromised biomechanical properties and the deposition of toxic residues (Mendes et al., 2007). Prior dehydration of ECM-based grafts is required for the safe and effective use of ethylene oxide, to enable sufficient diffusion of the reagent through the graft and provide for effective post-processing elimination (through aeration) of the toxic breakdown products ethylene glycol and ethylene chlorohydrin (Lomas et al., 2001; Binnet et al., 2012). Following preliminary investigations (unpublished) into dehydration methods, evaluation of ethylene oxide treatment of the decellularised nerve grafts was excluded from the study, since lyophilisation appeared to induce widespread structural disruption to the endoneurial tubules of the decellularised nerves, and desiccation at ambient temperature, appeared to irreversibly compromise rehydration potential.

The present study indicated that the effects of individual sterilisation methods on the properties of decellularised nerve ECM are strongly related to whether the method utilised ionising radiation (e.g., gamma radiation, E Beam) or oxidising agents (PAA in solution). Although there were no significant differences observed in the *in vitro* cytocompatibility of the variously treated decellularised nerves, or collagen/denatured collagen content, compared to non-treated samples, qualitative histological and immunohistochemical assessment revealed some changes to the ECM structure and basement membrane composition. Those treated with ionising radiation (gamma and E beam) appeared to retain the histoarchitectural features of the ECM, and positive antibody labelling of the main basement membrane constituents (laminin, fibronectin and collagen type IV). Treatment with the oxidising agent PAA resulted in disruption to ECM structures (primarily to the fine structure of the endoneurium), and an absence of observable antibody labelling for collagen type IV.

Previous studies have shown that disruption of ECM structures following PAA sterilisation occurs in a tissue-specific manner. For example, some adverse alterations in matrix structure were demonstrated in decellularised murine lung following PAA treatment (0.1% PAA in 4% ethanol for 2 h; Bonenfant et al., 2013). These changes included alterations to the structure of the alveoli in the central region of the lungs, with associated potential negative implications for the key tissue function of facilitating gaseous exchange. In contrast, a study on decellularised porcine temporomandibular discs found that PAA treatment (0.2% PAA in 4% ethanol for 6 h) did not induce significant structural alterations to the surface microarchitecture of the ECM (Matuska and McFetridge, 2015). The loss of structural integrity seen in the present study, particularly to the endoneurial region, may have negative implications for the capacity of the decellularised nerves to promote functional neuroregeneration. There is evidence that endoneurial tubules provide directional chemotrophic guidance and physical protection to individual neurons and migrating Schwann cells, therefore loss of defined tubule structure may be inhibitory to cell migration/growth across a defect site (Zochodne and Levy, 2005). Conversely, the more open extracellular matrix structure observed following PAA treatment could mitigate against tightly packed structures (such as the lamellar layers of the perineurium) physically impeding cellular infiltration. Intact endoneurial tubules may facilitate direct infiltration of Schwann cells and subsequent promotion of axonal regeneration through formation of Bands of Büngner (Gaudet et al., 2011). When endoneurial tubules are not directly aligned, as in any transection injury repaired by surgical means, it is probable that the structure of the endoneurium is at least partially remodelled (Witzel et al., 2005).

In contrast to the PAA treated samples, the structure of the peripheral nerve extracellular matrix appeared to be well preserved after gamma irradiation or E beam treatment. This differs to some published studies on soft tissues, such as that of Cao et al. (2013) which found that irradiation of freeze-dried aortas at 20 kGy caused widespread structural damage to the extracellular matrix, including the formation of small holes and a loss of luminal structure. Bonenfant et al. (2013) found that decellularised murine lungs irradiated at 60 Gy suffered significant structural damage, particularly to the alveolar region. A plausible explanation for the differing impact of exposure to irradiation seen in the current study compared to the previous studies could relate to the hydration state of the tissue during irradiation. Tissue structures and biomechanical properties have previously been found to be compromised in the lyophilised samples of tendon allografts and human-sized decellularised ovine liver, but preserved in the presence of water (Smith and Kearney, 1996; Kajbafzadeh et al., 2013). Where tissue scaffolds are lyophilised (or dried by another mechanism) prior to irradiation, gamma photons or free electrons are thought to interact directly with collagen chains that form the structure of the extracellular matrix, mediating the process of peptide chain scission and subsequent degradation of physical properties and loss of structural integrity (Bowes and Moss, 1962; Bailey et al., 1964; Smith and Kearney, 1996). In contrast, if tissues are kept immersed in a suitable solution (e.g., physiological saline or PBS), the photons and electrons interact with water molecules and induce radiolysis. This process yields free radicals (including hydrogen and hydroxyl free radicals) that interact with the collagens, predominantly causing the formation of additional crosslinks between collagen chains, which may act to add stability to the matrix structure (Bailey, 1967; Edwards et al., 2017). Since the nerves in this study were irradiated whilst immersed in PBS it is likely that the indirect interaction that favours formation of crosslinks predominated, facilitating preservation of the endoneurial tubules, as evidenced in the histological analysis.

The basement membranes of the peripheral nerve contain collagen type IV, which provides support and attachment for glycoproteins such as laminin and fibronectin (Gardiner, 2011). The universal retention of laminin and fibronectin demonstrated following sterilisation is highly promising when considering the ability of the sterilised scaffolds to support the infiltration of cells. Laminin in particular is known to be crucial for enabling the migration and attachment of Schwann cells within the endoneurial tubules, which in turn facilitate the processes of axonal regeneration and subsequent myelination. This is a crucial for progression toward functional restoration, and therefore underlies the importance of retaining such components within the decellularised nerves.

Previous studies in a range of decellularised tissues, have indicated that PAA treatment has a damaging effect on collagen type IV including decellularised human dermis (Hogg et al., 2015). A study comparing the presence of collagen type IV in decellularised porcine mitral valves, before and after treatment with 0.1% (v/v) PAA in PBS, demonstrated similar results to those observed in the present study, with a loss of positive antibody labelling of collagen IV following the PAA treatment (Granados et al., 2017). As yet, the nature of this damage and the mechanisms by which it occurs are not fully elucidated. It has been postulated that the loss of positive antibody labelling may reflect complete removal of the collagen type IV molecule, or be a result of changes in the structural conformation of the protein preventing binding (Luo et al., 2014). Since collagen type IV networks provide a structural base for other components of the basal lamina, such as laminin and fibronectin, which were retained following PAA treatment, complete degradation (or loss) of collagen type IV is unlikely. Instead, changes in conformation and/or surface chemistry may have occurred, preventing high affinity antibody binding and causing the observed loss of the positive signal. PAA is known to react with aliphatic side chains of amino acids, and this was proposed by Matuska and McFetridge (2015) as the likely mechanism behind changes in decellularised temporomandibular discs detected by Fourier Transform Infrared Spectroscopy. The PAA oxidative reactions may therefore explain the changes observed in this present study, at least partially. Additionally, peracetic acid treatment may have contributed to protein denaturation, as suggested in a study on sterilisation of decellularised murine lungs where increased levels of solubilised collagen IV components were detected in analysis by mass spectroscopy (Bonenfant et al., 2013). Protein denaturation may therefore have altered the antibody binding site on the collagen IV molecules in our study, although the 0.1% PAA solution in this case was at neutral pH in a buffered solution compared to the non-buffered and alcoholic solution used for the decellularised murine lungs (Bonenfant et al., 2013).

The implications of basement membrane collagen type IV changes on cellular interaction and promotion of neuroregeneration are unclear. Basement membranes are found in both the perineurium and the endoneurium of peripheral nerves, facilitating cellular attachment to, and interaction with, the extracellular matrix (Gardiner, 2011). Studies indicate that Schwann cells and the growth cones of regenerating neurons predominantly interact with laminin and fibronectin (Gardiner, 2011). However, a 2013 study found that rat Schwann cells directly interact with collagen type IV through a G-protein coupled receptor, Gpr126 (Paavola et al., 2014). This activates a cAMP-mediated decellularised signalling pathway and stimulates subsequent differentiation toward a myelinating phenotype. Furthermore, the study found that the disruption of this interaction by an induced mutation led to a loss of myelin basic protein expression. Whilst the interactions of human Schwann cells with collagen type IV are not well characterised, it is possible that myelination may be at least partially dependent on a specific interaction with collagen type IV. It is unknown whether the PAA-induced changes to collagen type IV seen here alter the binding site (structurally or chemically) of Gpr126 or an equivalent receptor in human Schwann cells. The possible effects on myelination remin unknown. The absence of positive antibody labelling of collagen type IV in the decellularised nerves following PAA treatment indicates that some kind of biochemical or structural change took place. It will therefore be crucial to answer the question of whether these changes affect cell attachment motifs and cellular interaction with the scaffold in future studies.

There was no detectable change in the level of denatured collagen following exposure of the nerve segments to either ionising radiation method, but a significant drop in denaturation transition temperature was determined. The presence of free radical induced cross-linking of the ECM did not therefore translate to an increase in resistance to thermal challenge. This divergence in the impact on the ECM may have been due to the effects of intermolecular peptide chain scission of the collagen fibrils being masked by newly formed cross-links stabilising, resulting in a tertiary structure sensitive to increased temperature but not releasing significant levels or size of peptide, as proposed by Edwards et al. (2017) in relation to decellularised and irradiated porcine flexor tendons. Whether the preservation of tubule formation or changes in thermal stability improves or inhibits the *in vivo* degradation and regeneration profile of decellularised nerves remains to be determined. The oxidising ability of PAA may not produce sufficient quantity of hydroxyl radicals to induce stable crosslinking of the collagen matrix, which could explain why disruption to the endoneurial structures was seen as well as a decreased collagen denaturation transition temperature.

Mechanical properties of decellularised tissue scaffolds can be affected by the interactions of sterilisation methods with the extracellular matrix structure. No significant difference was detected in mechanical properties of gamma irradiated or E Beam treated samples, in comparison to non-treated decellularised nerves. Contradicting data was produced regarding the mechanical properties of PAA-treated nerves, specifically between parameters reliant on physical dimensions (such as ultimate tensile strength, which takes into account of cross-sectional area) and those measured independently of such values (such as maximum load). PAA-treated nerves were found to have a significantly decreased ultimate tensile strength in comparison to native tissue; however, no such difference was seen in the maximum load. The structural alterations and increased “looseness” of the extracellular matrix, demonstrated histologically in nerves treated with PAA, is likely to result in an increased cross-sectional area. This means the force sustained per unit area of the PAA-treated tissue is lower than that of decellularised nerve, which may explain the reduction in ultimate tensile strength seen. A reduction in UTS could lead to physical failure of the graft if it is subjected to high tensile forces *in situ*, potentially increasing the chance of re-injury. The levels of stress required for failure to occur would likely be high enough to induce injury in axons irrespective of such changes in graft mechanical properties.

The change in ECM structure, and consequently the cross-sectional area, could also have contributed to the differences seen between the Young’s Moduli of decellularised nerves treated with PAA and those treated with gamma radiation. There was no significant difference between the Young’s Modulus of PAA treated decellularised nerves when compared to non-treated samples. Another possible contributing factor may have been the likely induction of crosslinking during the gamma radiation of the tissue in a “wet state.” It has been documented that alterations to the Young’s Modulus of a substrate can affect Schwann cell and neuronal phenotype *in vitro* (Rosso et al., 2017). It is difficult to predict how the tissue-scale mechanical changes, such as those seen here, will impact on the mechanical microenvironment experienced by individual cells as they interact with the ECM. As E Beam treatment induced no differences in any of the measured mechanical parameters when compared to all other groups (with the exception of the NBF and NaOH controls), it is unlikely to negatively affect the capacity of the decellularised nerves to support neuroregeneration or subsequent neuronal function. Although ionising radiation is a validated terminal sterilisation methods, the alterations to the physical properties of tissue scaffolds may still alter some cellular interactions, particularly in mechanosensitive cells such as neurons (Willits and Skornia, 2004). Further studies will be required to assess the cellular responses to the sterilised scaffolds, including those of the innate immune system (macrophages) which have a role in directing the transition from pro-inflammatory responses to anti-inflammatory responses and constructive remodelling (Badylak et al., 2008; Keane and Badylak, 2015).

## Conclusion

The evaluated sterilisation processes were found to affect the properties of decellularised nerves in differing ways. The thermal stability of the samples was impacted by all treatments, with a reduction in collagen denaturation temperature evident. Treatment with PAA induced structural disruption to the endoneurial tubule structures, which are thought to provide mechanical support and directional chemotrophic guidance to regenerating axons and accompanying Schwann cells. PAA treatment has the additional disadvantage of not being a truly “terminal” sterilisation method, requiring a further packaging step in a controlled sterile environment. Some alteration to the mechanical properties of decellularised nerves was also seen following gamma radiation; however, this was only significant when compared to the PAA-treated decellularised nerves. Decellularised nerves treated with E beam retained mechanical properties, extracellular matrix structure and a basement membrane composition similar to non-sterilised nerves, and therefore E beam is the terminal sterilisation method with the most potential for use with decellularised porcine peripheral nerves, out of those evaluated in this study.

## Data Availability Statement

The raw data supporting the conclusions of this article will be made available by the authors, without undue reservation.

## Author Contributions

JH and GW contributed to data acquisition, analysis, and drafting of the manuscript. PR, HB, LJ, and S-PW contributed to and critical revision of the manuscript. JH, HB, LJ, and S-PW contributed to conception and design. JH, PR, HB, LJ, and S-PW contributed to interpretation. The manuscript was written through contributions of all authors. All authors have given approval to the final version of the manuscript.

## Conflict of Interest

The authors declare that the research was conducted in the absence of any commercial or financial relationships that could be construed as a potential conflict of interest.
